# Tracking Molecular Shear at Metal Surfaces Using Enhanced Lamb Wave Scattering in Plasmonic Nanocavities

**DOI:** 10.1021/acs.nanolett.5c03363

**Published:** 2025-10-21

**Authors:** Alexandra Boehmke, Jonathan Bar-David, Sarah Sibug-Torres, Bart de Nijs, Alex B. Ferere, Nicolas Large, Jeremy J. Baumberg

**Affiliations:** † NanoPhotonics Centre, Cavendish Laboratory, 2152 University of Cambridge, J J Thomson Avenue, Cambridge CB3 0US, United Kingdom; ‡ Department of Physics and Astronomy, 414492 The University of Texas at San Antonio, San Antonio, Texas 78249, United States

**Keywords:** Lamb mode, surface acoustic wave, localized plasmon, Brillouin scattering, NPoM

## Abstract

Extreme plasmonic confinement to the nanoscale can be used to probe the configuration of molecules at metallic surfaces. Exploring low-frequency (*h*ν < *k*_B_ *T*) inelastic light scattering from molecular-monolayer-filled plasmonic nanocavities reveals additional low-frequency excitations not previously observed. We identify these as terahertz Lamb shear modes in the nanogap, exhibiting cross sections even larger than the surface-enhanced Raman scattering (SERS) of the vibrating molecules. Comparing different molecules and metals shows the influence on these Lamb modes of surface binding of the molecular monolayer as well as the strong impact of damping. The large occupation of such modes at room temperature implies their role across many fields, from electrochemistry, molecular electronics, and thermoelectrics to photocatalysis and sensing.

## Graphical abstract

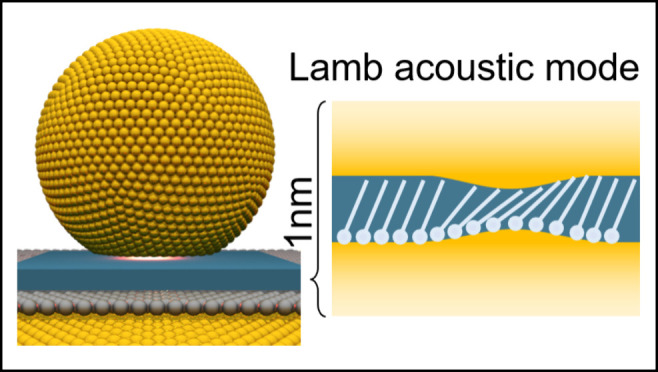

High-frequency acoustic modes in nanostructures give critical insight into the limits of high-speed mechanical dynamics. This influences fields from acousto-optic modulation in telecoms,^1^ optomechanical interactions for quantum devices,^2^ to mechanical forces in tribology.^3,4^ Conventional approaches using ultrasonic transducers become limited >10 GHz, while optical approaches access the terahertz but only for longitudinal strain modes spread over large areas.^5–7^ In dielectrics, the optical wavelength limits optomechanical interaction strengths, making the study of individual nanostructures challenging. Using plasmonic metal nanostructures that confine light below this diffraction limit gives opportunities to study acoustic properties of matter on the sub-10 nm scale.

Acoustic waves give a strain-modulated refractive index producing Brillouin scattering, but these are highly dispersive and thus not amenable to plasmonic enhancement.^8,9^ Besides these sound waves, acoustic modes in confined systems have cutoff frequencies providing a range of discrete resonances. One well-studied example is the acoustic radial breathing modes of spherical metal nanoparticles.^10–12^ By contrast, Lamb waves^13^ correspond to modes propagating along solid plates and also exist in soft layers sandwiched in between solid surfaces, such as glue layers in composites. These help detect delamination and cracking of thin composite plates up to gigahertz frequencies;^14^ however, they have never so far been experimentally observed in the terahertz domain.

Here, we examine the ultimate limit of Lamb modes in monolayer-thick molecular layers sandwiched between metal facets. Light is highly efficiently coupled into these nanogaps formed between an organic-monolayer-coated metal mirror and a nanoparticle deposited on top, forming a nanoparticle-on-mirror (NPoM) configuration (Figure 1a). Extensive works^15–17^ showed that such gold nanogaps support plasmonic waveguide modes, even when <1 nm thick. The resulting nanoscale optical modes penetrate the metal surface, probing the metal–molecule interface. Surface-enhanced Raman scattering (SERS), which scales as |**E**|^4^ for optical field **E** is >10^9^-fold enhanced in such nanogaps, providing detailed information about molecular configurations, binding, and dynamics at the metal surface. This is relevant to many areas, including electrochemistry, molecular electronics, organic optoelectronics and thermoelectrics, batteries, (photo)­catalysis, and sensing.

**Figure 1:**
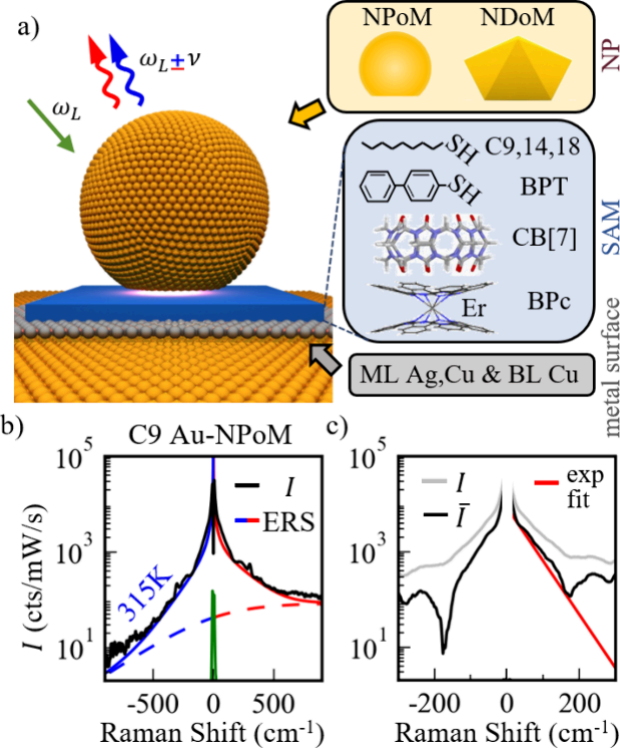
Terahertz SERS spectroscopy. (a) Schematic of the NPoM construct. (Boxes to the right) Control by nanoparticle shape (yellow), molecular SAM (blue, described in text), and surface atomic layer (gray) on the underlying Au mirror (ML, monolayer; BL, bilayer). (b) Low-frequency SERS spectrum from alkanethiol C9 SAM (log *y* scale) together with lines showing models of bosonic (solid) and fermionic (dashed) background from ERS. (c) Residual SERS before (gray) and after (black) the ERS is removed, showing a strong additional low-wavenumber signal, together with an exponential fit.

Using this metal–insulator–metal (MIM) sandwich at the heart of the nanostructure, we find that ultralow-frequency SERS uncovers nanogap terahertz acoustic modes through surface-enhanced Lamb wave spectroscopy. These modes are found to be heavily damped and dependent on the metal–organic interface binding. Using various molecular fillings, we show their universal behavior and identify their strong optomechanical interaction. Such modes contribute to the structural stability of nanogaps at ambient temperature and are relevant to their electrical, thermal, and optical properties.

Initially, individual nanogaps are examined using NPoMs with *D* = 60–100 nm diameter Au nanoparticles (faceted spherical) spaced *d* = 0.4–3 nm above a Au mirror by a self-assembled monolayer (SAM) of oriented molecules (Figure 1a). NPoMs trap light inside the nanogaps at specific wavelengths easily measured using dark-field scattering,^15,16^ with dominant plasmonic modes in the 700–900 nm range.^17^ Alternative nanodecahedron-on-mirror (NDoM) constructs offer consistent (111) Au triangular facets.^18^ A range of molecular SAMs are investigated (Figure 1a), by immersing the Au mirror in solutions (Methods in the Supporting Information) containing alkanethiols (C*n*−SH), biphenylthiol (BPT), barrel-shaped rigid cucurbiturils (CB­[*n*]), or bis-phthalocyanine (BPc, with Er^3+^ binding two planar organic cyanines). As well as these individual nanogaps, we create monolayer sheets of Au nanoparticles with similarly controlled gap size using molecular spacers (termed monolayer aggregates or “MLaggs”;^19^ Methods in the Supporting Information), which confine light inside many nanogaps.

Laser light at λ = 785 nm (100 μW) is focused onto well-separated individual NPoMs located by dark-field microscopy and the Raman-scattered photons detected across both Stokes and anti-Stokes energies (Figure S1). Using holographic angle-tuned filters and stabilized lasers allows Raman photons of <10 cm^–1^ separation from the laser to be isolated, providing access to terahertz modes (1 THz = 33 cm^–1^). Since nanoparticle shapes/facets vary, many different NPoMs are probed for each construct type (*N* > 100), and screened by cluster analysis.^20^

While the weak signal from flat Au consists of narrowband elastic laser scattering (Figure 1b, green), SERS from the NPoMs shows a broad response to both higher and lower energy as well as discrete lines from molecular vibrations (previously studied^15^). This broad response is believed to arise partly from optical scattering of free electrons in the metal.^20^ The exponential decay on the anti-Stokes side (Figure 1b, blue) allows an electron temperature to be extracted and used to fit and subtract this electronic Raman scattering (ERS). Its best fit (Figure 1b) corresponds to the expected Bose–Einstein expression^20^ (solid lines), *I*_Au_(ν) ∝ *n*_BE_(ν) + θ­(ν), where *n*_BE_ = [exp­{*h*ν/*k*_B_ *T*} – 1]^−1^ is the bosonic thermal population at energy *h*ν and θ = {0 (ν < 0), 1 (ν ≥ 0)}.

Once the ERS is removed, the remaining signal *I̅*(ν) is examined (Figure 1c). For alkanethiols, which possess weak Raman cross sections, vibrational peaks are minimal, and the signal *I̅*(ν) shows an exponential decay over more than 1 decade (Figure 1c, red). Even without ERS subtraction (Figure S2), this decay is strongly evident, atop the background (gray, with the thermal energy scale of >200 cm^–1^). The extra signal is seen on both Stokes and anti-Stokes sides (ruling out many instrumental artifacts) and is proportional to the ERS contribution, implying that it also arises from the enhanced optical field in the nanogap.

To identify the origin of this strong component, we study a wide range of samples (Figure 2). In almost all cases, the same exponential decay is seen (in both Stokes and anti-Stokes). It does not vary significantly in line shape for alkanethiols of increasing length from C9 to C18, which increase the nanogap from 1.5 to 2.8 nm (Figure 2a).

**Figure 2:**
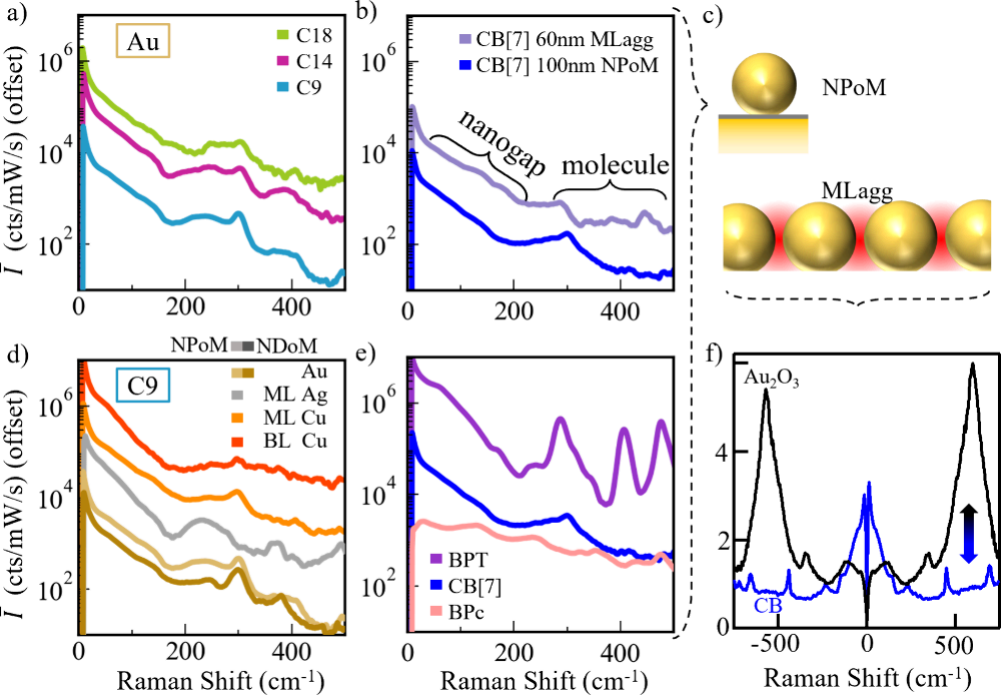
Terahertz SERS response after ERS subtraction. (a) Varying the alkanethiol length of Au NPoMs. (b) Varying the NP size and number of nanogaps containing the CB[7] molecule. (c) Schematic of the single nanogap (NPoM) and multiple nanogaps (MLagg). (d) Varying the metal on the mirror facet as well as Au nanoparticle shape between NPoM (light) and NDoM (dark). (e) Varying the type of molecule in the nanogap for Au NPoM. Curves are offset for clarity. (f) Normalized SERS spectra for both Stokes and anti-Stokes of MLagg in an electrochemical cell when cycling between +1 V (nanogaps filled with Au_2_O_3_) and −1 V (nanogaps filled with CB[7]).

Switching to the cucurbituril spacer (CB[7]) with a 0.9 nm gap gives a similar response (Figure 2b), varying little for different nanoparticle diameters (Figure S3). We also find similar results when obtaining data from MLagg samples with hundreds of nanogaps containing CB[7] (Figure 2b and c). While additional peaks are observed from molecular vibrations, the dominant component remains the low-frequency exponential contribution, which is independent of the laser power (Figure S4).

A third experiment uses C9 molecular SAMs but coats the template-stripped Au mirror with a single atomic monolayer of different metals using underpotential electrodeposition^21^ (Figure 2d). This changes the plasmonic modes little but perturbs the chemical anchoring.^21^ Examining Au, Ag, and Cu, which all bind thiols but at different lattice sites, shows that the exponential contribution is retained, with slightly varying decays. Adding a second atomic layer of Cu [to make a bilayer (BL)] also gives a similar response. Comparing different shaped Au nanoparticles for the upper nanogap facet also changes little, though the more consistent (111) facets of NDoMs reduce vibrational peak broadening (Figure 2d, brown light/dark lines).

We also compare molecules with different anchorings on the metal facets (Figure 2e). BPT (which has similar thiol binding to Au) shows similar results to C*n* alkanethiols, apart from extra vibrational peaks studied previously.^22^ Underlying these vibrations, the same exponential component is observed. CB[7] gives a very similar response. Although the CB[7] C=O portals bind to Au rather differently than thiols, they still give binding energies of >50% of S–Au bonds but attach to both top and bottom facets. Finally, we also create monolayers of BPc (which are consistent and reproducible, as quantified by dark-field spectroscopy^23^) that give 0.4 nm gaps but no direct chemical binding between Au and the molecule. In this case, the exponential signal is more than an order of magnitude weaker, with a very different energy dependence (Figure 2e, pink).

Finally, to prove that the origin of this exponential contribution depends on molecular filling, we use the MLagg as one electrode in an electrochemical cell with 50 mM potassium phosphate buffer and a CB­[*n*] solution. This allows a repeated cleaning and rescaffolding of the nanogap contents,^24^ which can be stripped of all organics and replaced with a plug of Au_2_O_3_, followed by rescaffolding with CB­[*n*] molecules. Clear switching is seen between the 600 cm^–1^ Au_2_O_3_ peaks and the 400 and 830 cm^–1^ CB peaks as the potential is cycled (Figure 2f). The low-frequency exponential contribution is absent for the oxide and appears only when CB­[*n*] fills the nanogaps.

To more quantitatively compare these results, we extract fit parameters *B* and ν_B_ of this additional terahertz exponential component, *I̅*_B_ = *B*e^–ν/ν_B_^. In all cases, the characteristic frequency ν_B_ ∼ 40 cm^–1^ is consistent (Figure 3a) and depends subtly on the surface metal layer that the thiol binds into, increasing by >70% between Ag and Cu monolayer-coated facets. By contrast, the strength of the signal decreases through this series of monolayer coverage metals (Figure 3b), as does the strength of the ERS signal. To account for in-coupling and out-coupling of the inelastically scattered photons, we thus normalize *B* by the ERS large-ν Stokes amplitude (Figure 1b), to give normalized amplitude *B̃*. Although the efficiency of ERS scattering might differ for different metals, the plasmonically confined light penetrates >15 atomic layers into the metal; therefore, a single surface atomic plane has little effect.^21^ Indeed, this is seen in the minimal (<10 nm) spectral shifts of the dark-field resonance wavelength. Comparing the normalized amplitude *B̃* shows it to be stronger from the Ag-coated faceted NPoM samples. On the other hand, this normalized amplitude varies little with the NP shape (Figure 3d) but is distinctively weaker (3-fold) for the longer C18 compared to C14 or C9.

**Figure 3:**
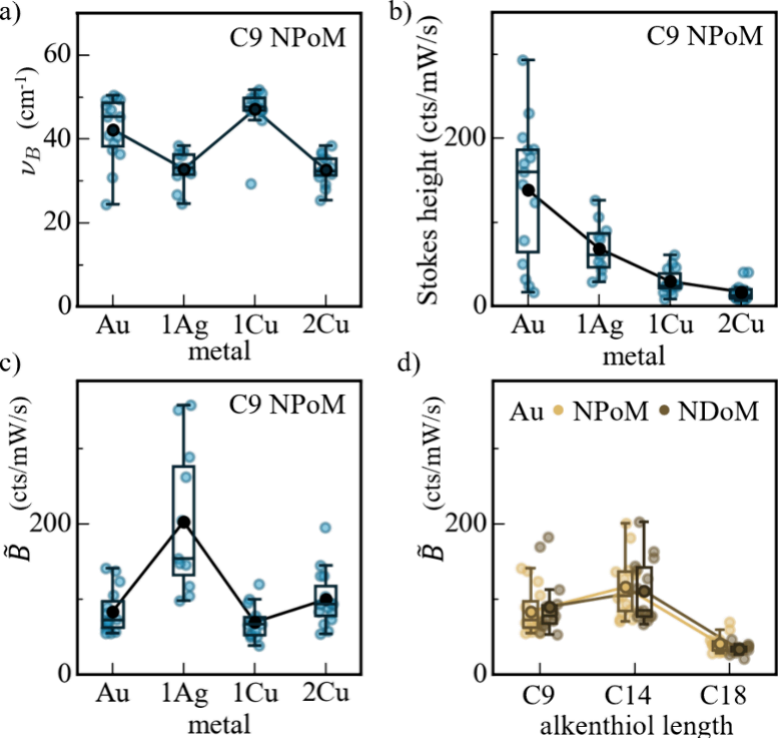
Comparison of exponential components in terahertz SERS. (a) Characteristic frequency ν_B_ and (b and c) signal amplitude vs surface metal atomic layer. In panel c, the amplitude is normalized by the ERS amplitude to give *B̃*. Points show raw fits; boxes show the full width at half maximum (fwhm); and lines show the mean. (d) Normalized amplitude vs increasing alkanethiol length.

We now consider the possible origins of this extra component for terahertz SERS. A similar contribution has been reported by Kamimura et al. for terahertz SERS from roughened metals without analytes,^25^ which the authors attributed to reduced long-range Coulomb screening and increased momentum transfer from plasmon resonances, without fully testing this hypothesis (previously they also suggested phonon modes of the NPs might be involved^26^). Several modes can contribute to terahertz inelastic scattering, including acoustic modes of the NPoM system, phonons, electronic excitations of Au, or quasi-elastic scattering (QES) from polarizability fluctuations of the SAM. QES does not appear to be the source, because no significant change is observed in characteristic frequency ν_B_ between highly polarizable (BPT) and much less polarizable (C9, C14, and C18) SAMs. The phonons of Au well-known from neutron scattering are not seen in inelastic scattering experiments and are not predicted to be Raman-active. Electronic excitations of Au would correspond to a modified ERS signal, but it is hard to see why a characteristic frequency ν_B_ ∼ 44 cm^–1^ ∼ 0.2 *k*_B_ *T* would emerge (heating can be ruled out, since no laser power dependence is found for ν_B_; Figure S4).

While Lamb (radial breathing) acoustic modes of 5 nm spherical Au nanoparticles are observed at ∼5 cm^–1^; the larger 80 nm diameters here would shift the frequencies below 10 GHz (far lower than observed) and would also depend on the nanoparticle shape, which is not seen here.^11^ To explore which other modes are Raman-active, we compute the acoustic modes of the MIM system and use them as input morphologies in finite element method (FEM) electrodynamic simulations (Figure S6 and Methods in the Supporting Information). We incorporate the recently developed Raman energy density (RED) formalism,^12^ which offers a powerful approach to identify spatial regions where acoustoplasmonic coupling gives rise to strong Raman scattering (Figure 4a). The RED represents the local electromagnetic energy density modulated by acoustic vibrations, linking the near-field plasmon–vibration interaction to the far-field Raman signal. The NPoM configuration supports localized surface plasmons whose interaction with acoustic vibrations gives rise to acoustoplasmonic Raman scattering.^27^ The RED approach spatially maps this coupling, revealing that the strongest Raman signals originate from the nanogap where surface plasmons and mechanical deformations overlap (Figure 4b and c and Figure S6a–c). FEM simulations of the strain distribution confirm that the Lamb modes are confined within the molecular layer (Figure 4d), decaying quickly inside the gold. The observed Raman response thus originates from deformations within the gap region, where plasmonic fields are co-localized.

**Figure 4:**
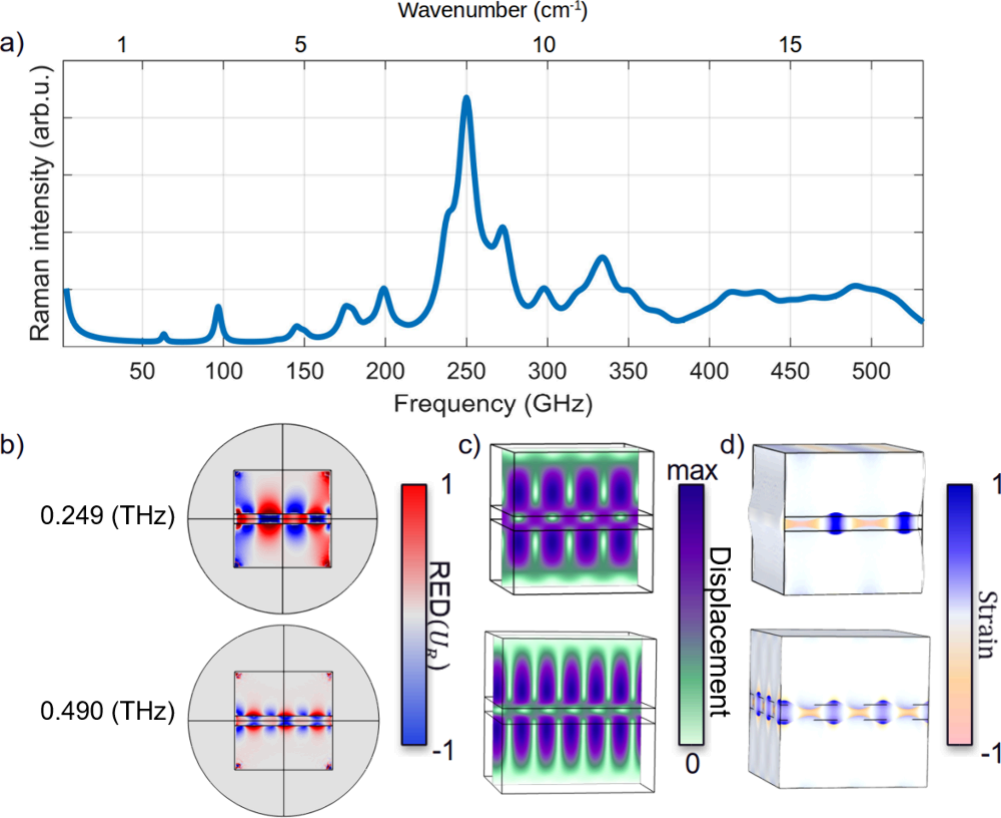
Modeling of surface acoustic waves in nanogaps. (a) Raman spectrum computed from over 800 vibrational modes in the Au–BPT–Au system. (b) RED calculated for the two Lamb modes at 0.249 and 0.490 THz. (c and d) Normalized displacement fields and strain, respectively. In panel d, the FEM model consists of a 1.1 nm BPT layer sandwiched between two gold slabs, each with a side of 5 nm.

Computing the Raman spectrum shows the vibrational modes with efficient acoustoplasmonic coupling, which significantly modulate the plasmonic near-field in the nanogap,^12,28^ with a 6.3 GHz bouncing mode identified as the dominant Raman-active mode (Figure S5). This mode, previously observed in pump–probe measurements,^29^ decays rapidly due to coupling with the substrate. Additional breathing (35.6 GHz) and quadrupolar modes (18.9 GHz) are still far below the terahertz experimental observations but correspond to the modes identified in RED.

To better understand their origin, we consider ideal modes of a thin soft layer between rigid plates, which are similar to those of a thin unsupported plate (Figure 5a–c). In the latter case, three types of modes exist, symmetric (*S*) or asymmetric (*A*) Rayleigh modes and a shear mode (*SH*), with the corresponding lowest modes *S*_0_, *A*_0_, and *SH*_0_ being acoustic-like. However, these acoustic-like modes are weak for MIM nanogaps as their strains remain unconfined. Both *SH* and *A* modes do not perturb the gap size (Figure 5b) and therefore cannot couple to the plasmon mode in the nanogap. On the other hand, *S*_*n*_ (Lamb) modes modulate the gap size *d*, thus changing the effective MIM refractive index ∝1/*d*. The long wavelength frequencies of the *S* modes (Figure 5c) are given by^30,31^

$$f_{S_{n}}=\left(\frac{c_{s}}{2d}\right)\left(2n-1\right)$$ 1

for positive integer *n*, shear velocity in the molecular layer $c_{s}=\sqrt{Y/\left(2\rho \left[1+\nu \right]\right)}$ ∼ 1100 m s^–1^ with estimates of molecular density ρ ∼ 1900 kg m^–3^, Young’s modulus^32^ *Y* ∼ 6 GPa, and Poisson ratio^33^ ν ∼ 0.32. The dominant mode for scattering is thus expected to be the *S*_1_ mode at *f*_S_1__ = *c*_s_/2*d* ∼ 0.5 THz, with higher order resonances at 1.5, 2.5 THz, etc. These are indeed close to the modes identified by RED and confirmed as Lamb modes by FEM simulations (Figure 4). This supports the low-frequency inelastic scattering observed as coming from coupled Lamb modes localized in the molecular monolayer. Differences in these predictions likely arise from the failure of a homogeneous slab to capture the behavior of the interacting molecular array within the layer.

**Figure 5:**
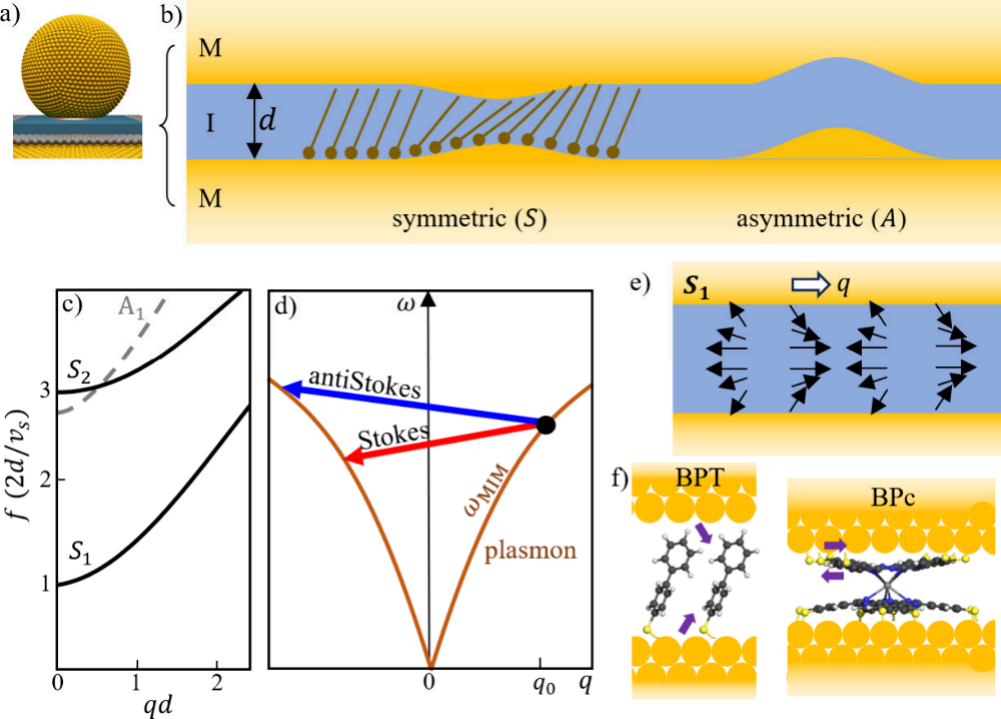
Surface acoustic waves in NPoM nanogaps. (a) NPoM constructs a nanogap that contains (b) a MIM waveguide that supports coupled Lamb modes that are symmetric or antisymmetric. (c) Lamb wave acoustic dispersion, *v*_s_ = shear velocity. (d) Plasmonic mode optical dispersion of MIM modes (line) with laser-pumped plasmon excited at 785 nm (circle) and arrows showing acoustic coupling to final plasmon states. (e) Displacement vectors for the lowest *S*_1_ mode showing alternating shear and compression of the molecule/metal interface. (f) Shear forces from thiol binding of BPT compared to shear slip for BPc (arrows).

Considering the planar facet region under the nanoparticle as a thin plasmonic MIM waveguide gives for the optical dispersion relation (Figure 5d)^15^ a plasmon in-plane wavevector *q*_0_ ≃ 2ε_g_(*d*|ε_m_|)^−1^ ∼ 0.2 nm^–1^ with gap permittivity ε_g_ ∼ 2 and metal permittivity ε_m_ ∼ −20 at 785 nm, for gap size *d* ∼ 1 nm. Since the Lamb mode disperses at *q* ∼ 1/*d* ∼ 1 nm^–1^ (Figure 5c), this broadens the inelastic scattering peaks (measurements are integrated over all directions). Emission of Lamb vibrations gives red-shifted emission (red arrow in Figure 5d). Plasmons can also absorb a Lamb vibration, blue shifting their emission (blue arrow), producing the observed anti-Stokes Raman scattering. Signals from higher order Lamb modes (Figure 4b) give smaller surface displacements and therefore weaker scattering.

The linewidth of surface-enhanced Lamb wave resonances depends on the Lamb vibration acoustic damping. From sparse experiments in the literature on organic molecules^34,35^ up to 100 GHz, acoustic damping is found to be proportional to (and similar magnitude to) the frequency (Figure 4a). The resulting broadening would thus give overlapping peaks.

This model thus provides an explanation of the observed terahertz SERS component observed. It explains why the decay would be dependent most strongly on the binding of the molecule to the metal (Figure 2e) rather than the exact molecular length or type (since covalent bonds have similar modulus). For molecules not bound directly to the gold, the shear involved in the Lamb mode displacement would be much more strongly damped (tracking the Au–molecule frictional interaction), giving very weak amplitudes. On the other hand, the molecular binding (≫*k*_B_ *T*) via thiol or carbonyl bonds gives distinct surface directionality (thiols set molecular tilts of ∼20–30°) and thus show strong Lamb mode scattering. It also suggests why metal type or nanoparticle size or shape would have little effect.

The Lamb mode frequencies *S*_*n*_ are set by the shear wave velocity through the SAM, which depends on the intermolecular interactions of the monolayer. Prior work^20^ shows that these interactions are mainly steric and thus expected to be similar in most SAMs but are extremely computationally expensive to model due to the need for realistic metal interfaces. Density functional theory (DFT) of SAMs show the single-molecule vibrations, and the Lamb mode can be considered as the coupling of these low-frequency molecular modes.^20^ Realistic inclusion of the metal surface in the DFT has been shown to be essential in capturing even single-molecule spectra, and the capability for simulating their coupling into Lamb modes is nascent so far. Additional broadening of the Lamb modes, however, can be expected from the range of intermolecular coupling expected in SAMs, which are never perfectly ordered (tilt direction unconstrained and SAM domain boundaries). The ability to also capture frictional dissipation in such coupling is even more challenging.

The identification of the low-wavenumber contribution (<50 cm^–1^) to inelastic light scattering from Lamb modes offers opportunities in measuring molecule–metal adhesion at the atomic scale. With typical facet diameters of 20 nm for 80 nm AuNPs,^36–38^ the plasmonic inelastic scattering is concentrated in the central 5 nm, thus measuring ∼200 molecules. Besides intermolecular coupling, these measurements also characterize friction at the atomic scale, opening up explorations of the influence of solvents, disorder, hydrogen bonding, and charge transfer. At the same time, thermal occupation of these low-wavenumber Lamb modes gives them a key role in storing energy, superposing waves of coordinated motions (as waves of wheat bending in a field). Besides their effect on light scattering, these coordinated motions will change electronic tunneling, charge hopping, reactivity, and catalysis as well as capping and damage processes.

In summary, confining light to the nanoscale enables exceptionally strong optomechanical coupling between surface plasmons and acoustic Lamb modes of soft organic monolayers confined in metal–insulator–metal nanogaps. Changing NP geometry, molecular attachment, gap morphology, optical power, tuning of plasmon by NP size, and molecular bonding suggests how coupling leads to enhanced low-frequency inelastic light scattering, with amplitudes comparable to those in conventional molecular vibrational SERS. This terahertz response is intrinsically linked to the nanogap optomechanical dynamics and the molecular–metal interface. The Raman energy density reveals that specific acoustic modes generate localized electromagnetic field modulations strong enough to produce detectable Raman signals. This enables the rational design of plasmonic nanocavities for enhanced acoustoplasmonic interactions. With the bridging of the conceptual and energy separation between Brillouin and Raman scattering, this work opens the way to new regimes of nonlinear optomechanical interactions, nanoscale tribology, and nanoscale quantum technologies. The ability to probe shear forces and dynamic bonding at the molecule–metal interface offers a powerful characterization tool at the molecule–metal interface critical for a wide range of nanoscale applications and charts a path toward engineering plasmonic platforms for tailored vibrational control at the nanoscale.

## Supplementary Material



## Notes

NPoM samples with different SAM molecules and their SERS (Figure S1), SERS spectra before/after subtraction of ERS and Lamb mode (Figure S2), SERS spectra for BPT Au NPoM with different NP sizes (Figure S3), power dependence of raw SERS spectra for 80 and 100 nm Au NPoMs with BPT (Figure S4), acoustic Stokes Raman spectrum from a NPoM system (Figure S5), modelling of surface acoustic waves in NPoM and MIM nanogaps (Figure S6) , and Methods (PDF)
