# Supplementary material for: Tracking Molecular Shear at Metal Surfaces Using Enhanced Lamb Wave Scattering in Plasmonic Nanocavities
Source: Nano Lett. 2025 Oct 21;25(44):15817–23. doi: 10.1021/acs.nanolett.5c03363 (PMC12593316; doi:10.1021/acs.nanolett.5c03363)
Supplement: Supplementary file 1 [file nl5c03363_si_001.pdf]

Supplementary Information

Tracking Molecular Shear at Metal Surfaces using Enhanced Lamb Wave Scattering in Plasmonic Nanocavities

Alexandra Boehmke[1], Jonathan Bar David[1], Sarah Sibug-Torres[1], Bart de Nijs[1], Alex B. Ferere[2], Nicolas Large[2], Jeremy J. Baumberg[1*]

[1] NanoPhotonics Centre, Cavendish Laboratory, J J Thomson Avenue, University of Cambridge, CB3 0US, UK
[2] Department of Physics and Astronomy, The University of Texas at San Antonio, San Antonio, Texas 78249, USA
* email: jjb12@cam.ac.uk

Contents:

Figure S1. NPoM samples with different SAM molecules, and their SERS.
Figure S2. SERS spectra before/after subtraction of ERS and Lamb mode.
Figure S3. SERS spectra for BPT Au NPoM with different NP sizes.
Figure S4. Power dependence of raw SERS spectra for 80 and 100nm Au NPoMs with BPT.
Figure S5. Acoustic Stokes Raman spectrum from an NPoM system.
Figure S6. Modelling of surface acoustic waves in NPoM and MIM nanogaps.
Supplementary Section Methods

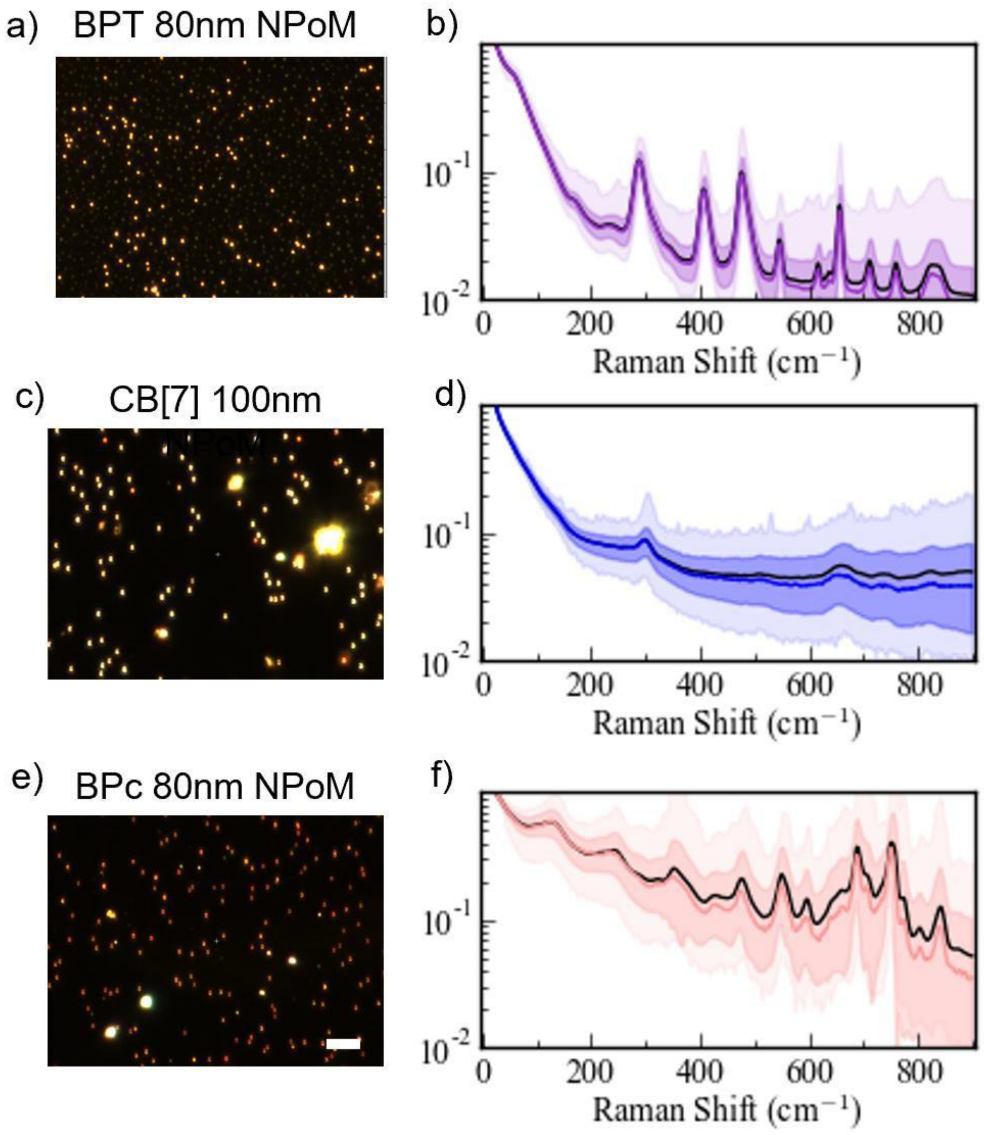

Figure S1. NPoM samples with different SAM molecules, and their SERS. (a,c,e) Dark field scattering images of typical sample regions, scale bar is 100µm. (b,d,f) SERS spectra from >100 NPoMs for each sample, showing mean spectrum (black), most typical spectrum from cluster analysis (bold), and range of spectra within the central cluster (darker region).

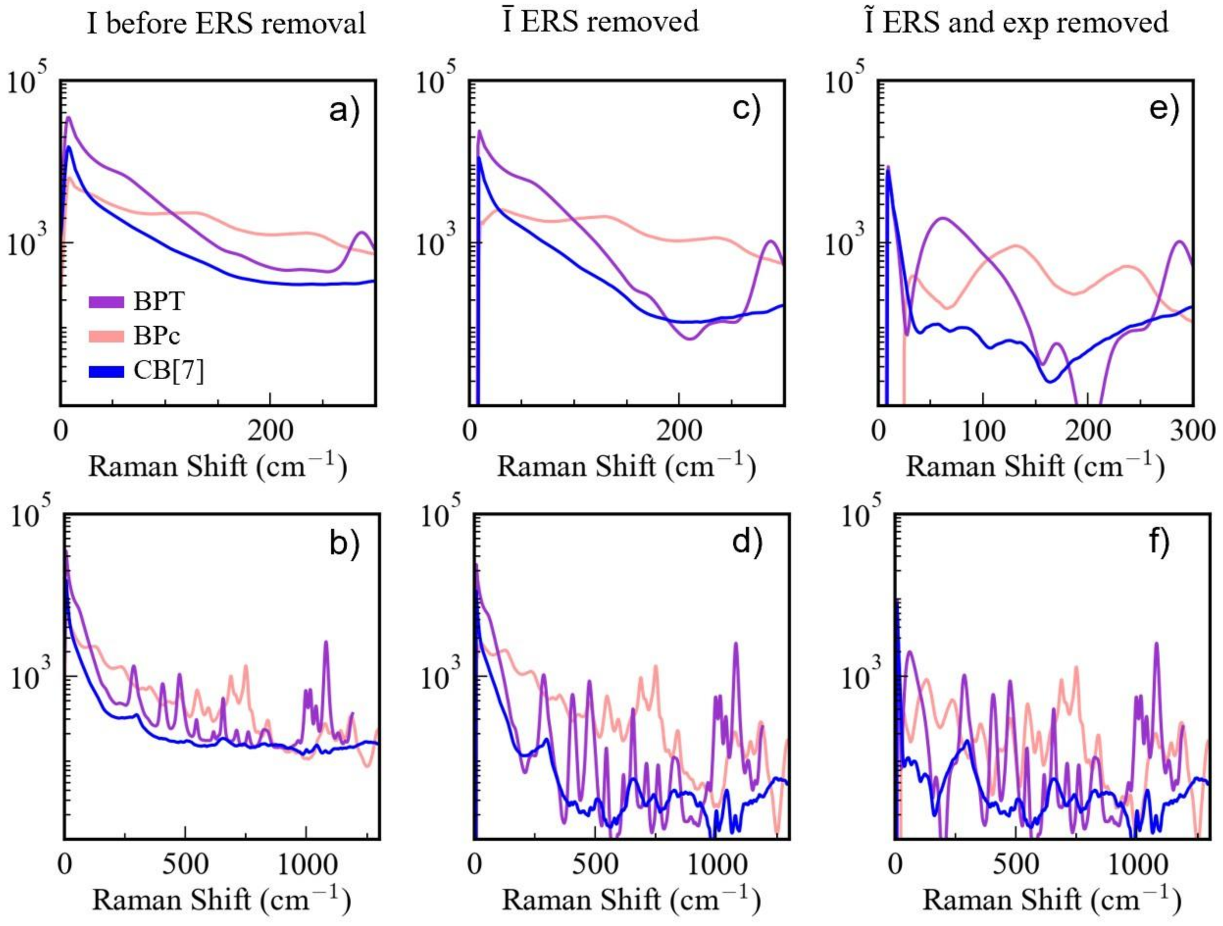

Figure S2. SERS spectra before/after subtraction of ERS and Lamb mode. (a-e) SERS spectra on log y-scale, for BPT, BPc and CB[7] molecular monolayers, (a,b) raw data before ERS subtraction, (c,d) after ERS fit and subtraction, and (e,f) after finally removing the fitted exponential Lamb mode. Low frequency range in (a,c,e) and wider range in (b,d,f). BPT and BPc use 80nm NPoMs, CB[7] with 100nm NPoMs.

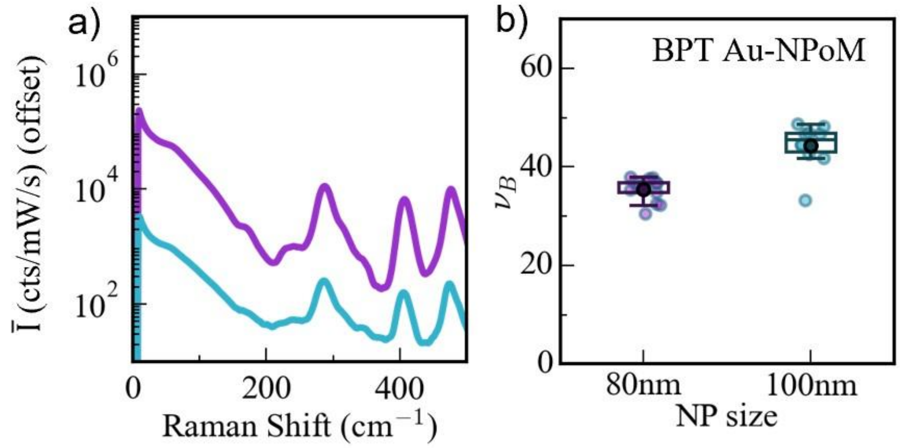

Figure S3. SERS spectra for BPT Au NPoM with different NP sizes. (a) ERS subtracted SERS spectra for 80nm NPs (purple) and 100nm NPs (blue). (b) Fit Lamb mode exponential decay rate for different NP sizes.

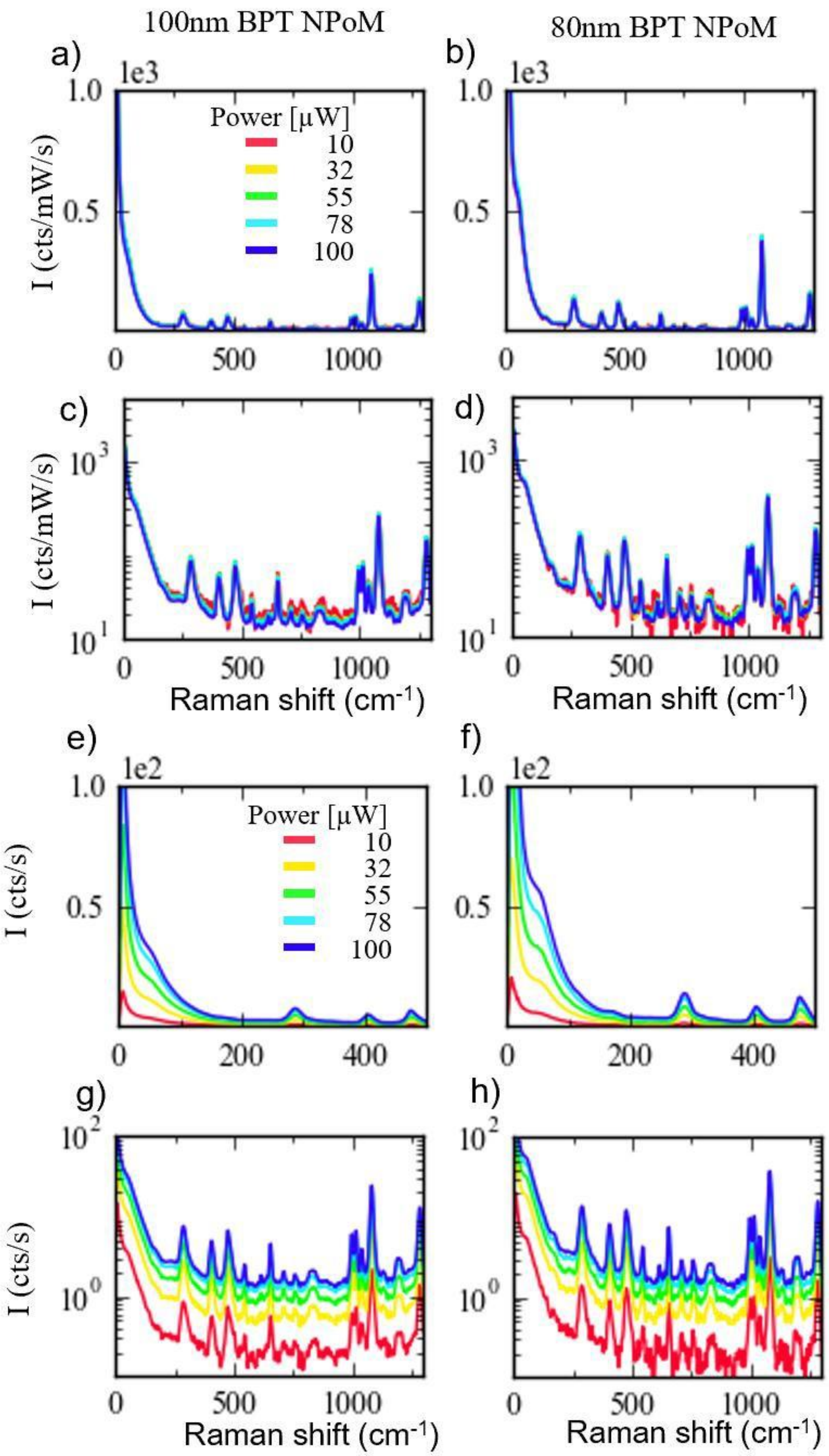

Figure S4. Power dependence of raw SERS spectra for 80 and 100nm Au NPoMs with BPT. (a,b,e,f) linear y-scale, (c,d,g,h) same data on log y-scale. Note different ranges on x-scale. (a-d) SERS normalised by laser power, (e-h) unnormalised SERS spectra. In all cases the low energy exponential Lamb mode is observed, together with a low frequency BPT vibration.

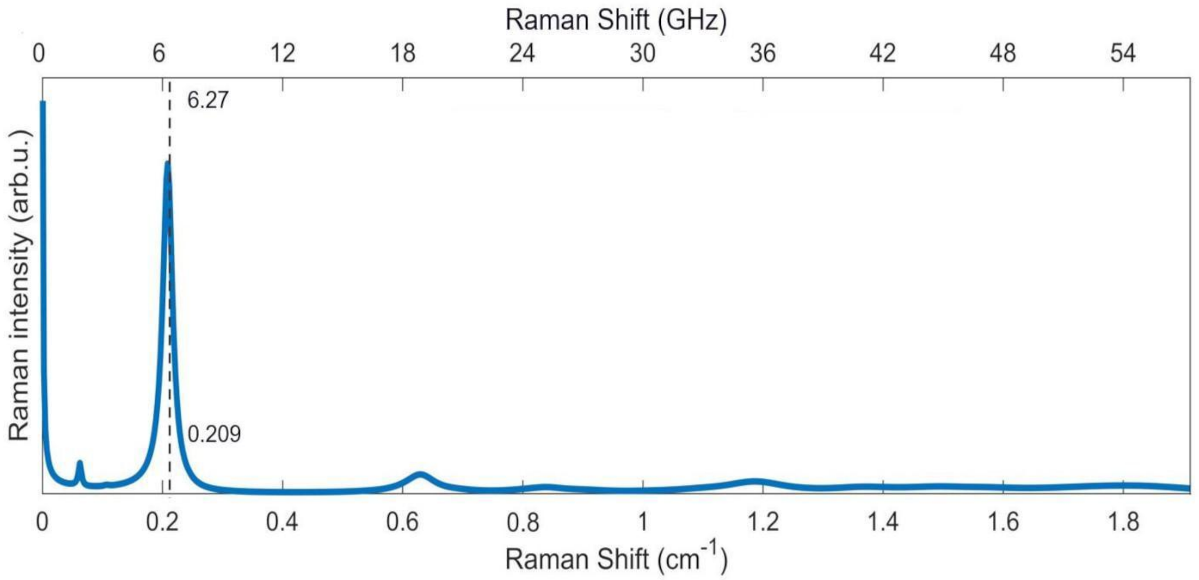

Figure S5. Acoustic Stokes Raman spectrum from an NPoM system under 642 nm optical excitation. The spectrum is computed from the contribution of ∼300 vibrational modes below 58 GHz using the RED formalism.

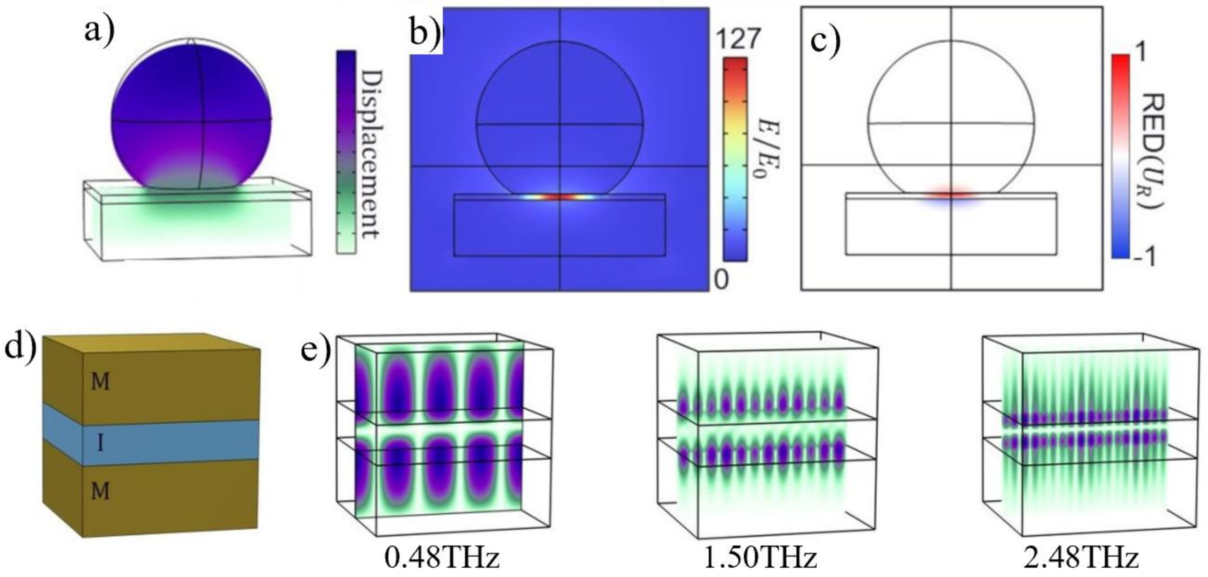

Figure S6. Modelling of surface acoustic waves in NPoM and MIM nanogaps. (a) Displacement associated with bouncing mode at 6.3 GHz. (b) Plasmonic near-field distribution for excitation at the main plasmonic coupled mode. (c) Raman Energy Density (RED) calculated for the bouncing mode shown in (a) and plasmon field in (b). (e) MIM model mechanically analogous to NPoM nanogap, supporting (f) Lamb waves for f_{S1} and higher order resonances. Displacement fields are normalized.

Supplementary Section Methods

Sample Preparation

The flat Au(111) surface is prepared by a template-stripping method. Approximately 100 nm of Au is evaporated at a rate of ~1.0 Å/s onto a Si wafer (Si-Mat) with typical roughness < 3 Å. Pre-cut 10 x 5 x 0.5-mm glass slides (UQG Optics) are then attached to this surface with UV glue (Norland 81) and cured with UV light (364 nm) for 35 minutes. The glass pieces can then be removed by cutting the perimeter with a blade, exposing a smooth Au surface with rms roughness < 0.2 nm.

Molecules from Sigma-Aldrich) were used as purchased. A SAM is formed on the Au surface by submerging the Au-coated glass into a 1 mM solution of the molecular crystal powder dissolved in anhydrous ethyl alcohol (Sigma-Aldrich >99.5%) for 16-24 hours. They are then rinsed with the same solvent and dried in N_2. Colloidal 80 nm or 100 nm diameter nanoparticles (BBI Solutions) are drop cast onto the SAM-covered surface, then rinsed after 30 s with deionized water, and N_2 dried. If sparse deposition occurs, the final step is repeated with a ratio of 10:60 µL $NaNO_3$ to nanoparticle solution.

MLaggs are created using the protocol in ref. [24]. Briefly 500 µL of chloroform ($CHCl_3$) is added to an Eppendorf, followed by 500 µL of 100 nm gold (BBI Solutions) nanoparticles. 100 µL of 1 mM cucurbit[5]uril (CB[5]) solution is then added and shaken for ~1 min to initiate aggregation. The mixture is left to settle so that the immiscible $CHCl_3$ and aqueous phases separate and the aggregated Au NPs move to the interfaces (chloroform-aqueous and aqueous-air). The aqueous phase is washed with three 300 µL aliquots of DI water to dilute the citrate salts and other supernatants, and then concentrated by careful removal of the aqueous phase to form a ~5 µL aggregate droplet floating on the $CHCl_3$. The droplet is deposited onto a pre-cleaned gold coated silicon substrate (100 nm Au, 5 nm Cr, on silicon) and left to dry. Once dried, the resulting Au NP multilayer aggregate (MLagg) is rinsed with DI water and dried with N_2. For their electrochemical regeneration, the samples are immersed in an electrochemical flow cell. This cell is fabricated from 10:1 PDMS:cross-linker, and its geometry adjusted to include a well, inlet and outlet, as well as inserts for electrodes. The three-electrode amperometric system includes a Pt counter electrode and Ag/AgCl reference electrode. The samples are used as the working electrode, contacted with Cu tape. The sample is then immersed in the flow cell with 1 M potassium phosphate buffer, pH 7.5. A constant potential of 1.5 V is applied for 60 s, followed by a rinsing step. Subsequently, the samples are immersed in 1 M potassium phosphate buffer and 1 mM CB[5], and a potential of -0.8 V is applied for 15 s. Finally, the samples are rinsed with DI water.

Spectroscopic Measurements

A custom system was built to measure SERS down to 5 cm^{-1} (Fig. 1a). A 785-nm diode laser (Integrated Optics, Matchbox Model 785L-21A) with external thermoelectric cooler (Integrated Optics, AM-H9) is collimated before passing through a pair of volume holographic grating (VHG) clean-up filters (OptiGrate BPF-785, FWHM < 0.12 nm) mounted on manual rotation stages with micrometers (ThorLabs) for angle tuning. The beam is coupled into a microscope (Olympus BX53M) fitted with a high-NA objective (Olympus LMPFLN100xBD, 0.8 NA) suitable for both SERS and darkfield spectroscopy. A halogen lamp is coupled through a darkfield mirror cube into the objective to measure the NPoM scattering spectrum for sample characterization. An x-y motorized stage (Prior Model PS3J100/D) enables automated measurements of many hundred single particles. Backscattered light is collected by the objective and passed through a series of three VHG notch filters (OptiGrate BNF-785, FWHM < 0.6 nm, OD3) separated by irises to minimize the collection of spurious scattering. The notch filters reject the narrow laser line while transmitting Raman scattering down to ±5 cm^{-1} to a single-grating spectrometer (Horiba Triax 550, 600l/mm grating) coupled to a 2048 x 512-pixel front-illuminated CCD (Andor Newton Model DU940P-FI).

Optomechanical Simulations

The acousto-plasmonic (*i.e.* optomechanical) simulations were performed in COMSOL Multiphysics (finite elements method, FEM) and following the theoretical formalism detailed in ref. [12].

FEM Models: The NPoM systems are modelled as (1) a 60 nm Au sphere with the outer 1/6 of its radius being faceted and placed on top of a 75×75 nm Au mirror and spaced by a 2 nm BPT layer; (2) a 1.1 nm BPT layer sandwiched between two gold slabs, each with a side length of 5 nm. Modes are obtained in a periodic MIM structure in which the BPT layer is approximated as a continuous elastic medium. Floquet boundary conditions are applied along the horizontal axes of the unit cell to approximate an infinite periodic structure, while low reflecting boundary conditions are used on the top and bottom gold surfaces to approximate a semi-infinite domain in the z-direction. This configuration reduces computational requirements while accurately representing the mechanical behavior of the NPoM structure within the gap region.

For the electrodynamic calculations, the dielectric permittivity for gold was taken from Johnson and Christy[37] and a constant refractive index of 1.45 used for the BPT layer. Within the gap region, sub-nanometer mesh elements were used to resolve the electric field with larger elements on the outer gold mirror and NP surface. The NPoM was illuminated by a uniform plane wave at normal incidence. The system is surrounded by 300 nm thick perfectly-matched layers (PMLs).

For the acoustic calculations the bottom plate of the gold mirror was mechanically fixed with all other surfaces using free boundary conditions. Vibrational modes were calculated using COMSOL's eigenfrequency solver with the maximum displacement limited to 2 nm (gap size) to prevent meshing errors during mesh displacement. The displacement amplitude was normalized as a function of vibrational frequency ($\sqrt{f_1 / f_n}$) using the first physical vibration mode. These displacements were then used to deform the mesh of the previous optical simulations to calculate the electric field modulation induced by each vibrational mode. The longitudinal and transverse wave speeds for gold were taken at 3082 and 1145 m/s, respectively, with a density of 19,700 kg/m^3.[38]

The RED calculations use a damping factor, which increased as a function of vibrational frequency with the deformation potential and interband transition values taken as –0.8 eV and 2.4 eV for gold, respectively[38]. The temperature was assumed to be a constant 300 K.
